# Whole-Exome Sequencing Enables Rapid Determination of Xeroderma Pigmentosum Molecular Etiology

**DOI:** 10.1371/journal.pone.0064692

**Published:** 2013-06-03

**Authors:** Oscar Ortega-Recalde, Jéssica Inés Vergara, Dora Janeth Fonseca, Xiomara Ríos, Hernando Mosquera, Olga María Bermúdez, Claudia Liliana Medina, Clara Inés Vargas, Argemiro Enrique Pallares, Carlos Martín Restrepo, Paul Laissue

**Affiliations:** 1 Unidad de Genética, Escuela de Medicina y Ciencias de la Salud, Universidad del Rosario, Bogotá, Colombia; 2 Departamento de Dermatología, Universidad Autónoma de Bucaramanga, Bucaramanga, Colombia; 3 Unidad de Dermatología, Clínica Carlos Ardila Lulle, Bucaramanga, Colombia; 4 Departamento de Biología Molecular, Genética Molecular de Colombia, Bogotá, Colombia; 5 Universidad Industrial de Santander, Bucaramanga, Colombia; Ohio State University Medical Center, United States of America

## Abstract

Xeroderma pigmentosum (XP) is a rare autosomal recessive disorder characterized by extreme sensitivity to actinic pigmentation changes in the skin and increased incidence of skin cancer. In some cases, patients are affected by neurological alterations. XP is caused by mutations in 8 distinct genes (*XPA* through *XPG* and *XPV*). The XP-V (variant) subtype of the disease results from mutations in a gene (*XPV*, also named *POLH*) which encodes for Polη, a member of the Y-DNA polymerase family. Although the presence and severity of skin and neurological dysfunctions differ between XP subtypes, there are overlapping clinical features among subtypes such that the sub-type cannot be deduced from the clinical features.

In this study, in order to overcome this drawback, we undertook whole-exome sequencing in two XP sibs and their father. We identified a novel homozygous nonsense mutation (c.897T>G, p.Y299X) in *POLH* which causes the disease. Our results demonstrate that next generation sequencing is a powerful approach to rapid determination of XP genetic etiology.

## Introduction

Xeroderma pigmentosum (XP) is a rare autosomal recessive disorder which affects 1/250.000 to 2.3/million individuals in Western countries [1], [2]. Higher incidence has been reported in specific regions, such as Japan (1∶20.000) and Tunisia (1∶10.000) [3], [4]. XP patients are affected by extreme sensitivity to actinic pigmentation changes in the skin and increased incidence of skin cancer. Some patients display neurological alterations such as loss of intellectual functioning, progressive hearing loss, neuromuscular degeneration and tumors of the central nervous system [5], [6]. XP is caused by mutations in 8 distinct genes (named *XPA* through *XPG* and *XPV*), which encode proteins that participate, excepting for XPV, in the nucleotide excision repair (NER) molecular cascade [2]. The NER system is responsible for repairing DNA damage resulting from environmental factors, such as UV radiation and carcinogenic chemical molecules [7]. This multistep mechanism excises and eliminates damaged nucleotides followed by filling in of the consequent gap, using the complementary DNA strand as a template. Consequently, patients carrying NER molecular dysfunctions exhibit a wide spectrum of phenotypic features, including XP [8].

The XP-V (variant) subtype of the disease results from mutations in a gene (*XPV*, also named *POLH*) that is not involved with the NER pathway. *POLH* encodes for Polη, a member of the Y-DNA polymerase family, which is implicated in the synthesis of DNA after injury (translesion synthesis process) [9]–[11]. Indeed, in XP-V cells *POLH* mutations reduce the ability to replicate DNA after UV exposure [12], [13].

Although the presence and severity of skin and neurological dysfunctions differ between XP subtypes, there are overlapping clinical features among subtypes such that the sub-type cannot be deduced from the clinical features.

In this study, in order to overcome this drawback, we undertook whole-exome sequencing in two XP sibs and their father. We identified a novel homozygous nonsense mutation (c.897T>G, p.Y299X) in *POLH* which causes the disease. Our results demonstrate that next generation sequencing is a powerful approach to rapid determination of XP genetic etiology.

## Materials and Methods

### Patients

**Figure 1:**
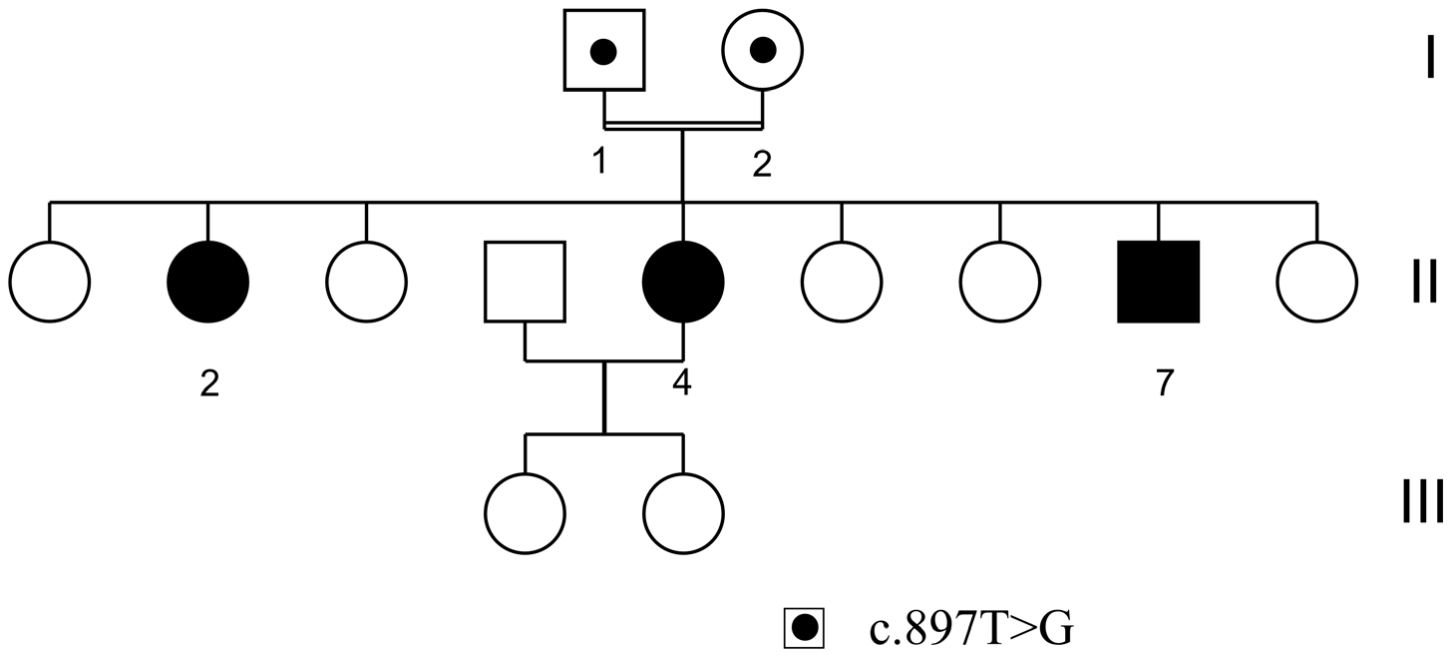
Pedigree of the XP family. Black symbols refer to affected individuals. Black dots into first generation individuals symbols (I:1 and I:2) represents the *POLH* c.897T>G mutation at heterozygous state.

Patients (P1-II:2, P2-II:4) are sibs who attended the Dermatology Unit of the Carlos Ardila Lulle Clinic of Bucaramanga (Colombia) (**Figure 1**). We could not obtain clinical data and biological samples from patient 3 (P3-II:7) but his family indicated that he is affected by a similar phenotype (see below). This study has been approved by the Ethical Committee at Universidad del Rosario and was conducted according to the Declaration of Helsinki Principles. Patients and their parents (C1-father, C2-mother) provided a written consent form to participate in the study, which includes an authorization to publish these case details.

The patients’ parents were reported to be consanguineous (first cousins). P1 is a 38-year-old female who presented numerous sun*-*induced skin changes since infancy. At the age of 21 years she presented a unique skin lesion, located on the dorsal region of the nose, which was diagnosed as a squamous cell carcinoma. Since then, 4 additional tumors (basal cell carcinomas) have been diagnosed and surgically resected. No antecedents of melanoma or further neoplasias were recorded. At the age of 37 years physical examination of the skin displayed: Fitzpatrick phototype III, xerosis, multiple telangiectasias and hyper and hypo pigmented lenticular macules (2 mm–5 mm). These lesions were especially located on UV exposed regions, such as face, upper limbs, feet, legs and upper thorax. Additionally, she displayed plaques of keratosis located on the face, arms and legs. Palm and soles, scalp, mucosae and nervous system were unaffected.

P2 (P1′s sister) is a 36-year-old individual affected, since early infancy, by significant photosensitivity related to the occurrence of skin pigmented lesions, which develop on the sunlight exposed areas of the body. Between 31–36 years of age, six basal cell and one squamous cell carcinomas were diagnosed. Physical examination of the skin, performed at 36 years of age, displayed similar findings to those observed in her sister, excepting for the presence of 3 lesions suggestive of basal cell carcinoma.

### Exome Capture and High-throughput Sequencing

Genomic DNAs from patients (P1 and P2) and their parents (C1 and C2) were extracted from whole blood samples using standard procedures. Three micrograms of genomic DNA from P1, P2 and C1 was made up to 130 µl with nuclease free water and sonicated to fragment DNA into size ranging between 100 base pairs (bp) to 200bp (Covaris S220). The size distribution was checked by running an aliquot of the sample on Agilent HS Bioanalyzer Chip. The fragmented DNA was size selected using Agencourt AMPure XP SPRI beads (Beckman Coulter). Subsequently, genomic DNA libraries were constructed according to the SureSelect Target Enrichment System protocol using SureSelect Library prep kit (Agilent). DNA was subjected to a series of enzymatic reactions that repair frayed ends, phosphorylate the fragments, add a single nucleotideA overhang and ligate adaptors. After ligation, PCR amplification (5 cycles) was performed to enrich the adaptor-ligated fragments. The prepared libraries were then kept for hybridization as outlined in SureSelect Target Enrichment System for SOLiD 5500 Multiplexed Sequencing protocol. Hybridized library fragments were isolated by magnetic capture using Magnetic Streptavidin coated beads. PCR amplification (8 cycles) was carried out to amplify the captured library and cleaned up using AMPure XP beads (Beckman Coulter). An aliquot of the captured library was run on Agilent High Sensitivity Bioanalyzer Chip (Agilent).

### Reference Genome and RefSeq Database

We used the human reference genome GRCh37/hg19 for mapping exome-sequencing (Exome-Seq). The RefSeq sequence database downloaded from NCBI on 12 May 2011 was used as our gene model and for determining aminoacid substitutions.

### Exome-Seq Processing-Read Mapping, Variant Calling and Effect Determination

Raw data was obtained from the SOLiD5500×l sequencer and subjected to quality check and filtering using SAET (SOLiD Accuracy Enhancement Tool, LifeScope suite). The high quality filtered data were aligned with human reference genome and variants were called against (LifeScope suite). Then, variations (vcf. file) were transferred to local linux machine. Using snpEFF (sourceforge) the filtered variations were classified, based on their function, into distinct categories such as synonymous, non-synonymous, nonsense, missense, insertions, deletions and splice variations.

Results were further compared with the dbSNP database combining NCBI version 129 to db 137. This helped to identify novel variations and potential deleterious effects using snpSIFT/snpEff. Finally, the annotated variations were further reviewed manually. Exome sequencing assays were performed at Genotypic Technology (Bangalore, Karnataka, India).

### *POLH* Direct Sequencing

In P1, P2, C1 and C2 the exon 8 coding sequence of *POLH* (ENST00000372236) was amplified using exon-flanking oligonucleotides. Amplicons were purified using shrimp alkaline phosphatase and exonulease I, and sequenced with internal primers. Primer sequences and PCR conditions are available on request. The new sequence data has been deposited in the NCBI-dbSNP database under the accession number rs190423114.

## Results

We generated 21 GB data for 3 samples for each individual as paired-end, 75 bases forward and 35 bases reverse, and about 76–85% (38.90–43.51 Mb in length) of the targeted bases were covered at 20X coverage, which sufficiently passed our thresholds for calling SNPs and short insertions or deletions (indels). The bases with quality scores above 20 (99% accuracy of a base call) represent over 79–86% of total sequence data.

Exome-Seq processing showed that patients and C1 are respectively homozygous and heterozygous for the *POLH* c.897T>G (p.Y299X) mutation. Direct sequencing of *POLH* exon 8 confirmed these findings. We did not find potential etiological non-synonymous variants in any of the other XP genes.

## Discussion

At present, XP patients are classified into eight distinct subtypes depending on the occurrence of mutations in specific genes. However, it has been described that ∼6% of XP cases do not carry mutations in *XPA*-*G* or *XPV*, which suggest potential promoter regulatory variants as well as undiscovered etiological genes that could be identified using direct sequencing or NGS technology [5]. In this context, it would be interesting to sequence, via NGS, XP patients from previous reports who lack mutations in *XPA* through *XPG* and *XPV* genes.

Although the majority of subtypes implicate dysfunctions of proteins which participate in the NER molecular pathway, overlapping clinical features among patients have been observed. Furthermore, the clinical presentation of XPV can be similar to that observed in patients carrying XP-NER gene mutations. For instance, although most XP-V, XP-C and XP-E patients (which represent in Europe and the United States 40–58% of all XP cases) lack severe sunburn reactions, some cases display extreme phenotypes [2], [14], [15]. Mutations in XPC and XPE subtypes, which usually do not lead to neurological disease, can present central nervous system abnormalities due genetic and environmental modifier factors [2], [16], [17]. XPV patients (who are rarely affected by neurological abnormalities) can exhibit skin injuries that vary considerably in severity [18].

In this context, selection of a particular candidate gene for direct sequencing remains difficult. In addition, direct sequencing of all XP coding regions from genomic DNA might be challenging as they encompass more than 100 exons (∼25 kilobases). Whole-exome sequencing has led to the identification of a considerable number of pathogenic mutations in monogenic mendelian disorders, including skin pathologies [19]–[22]. This approach is especially useful for investigating recessive pathologies, as comparative analysis of coding sequence variations between affected and non-affected individuals enable the efficient identification of homozygous causative mutations [23].

Clinically, P1 and P2 are affected by a mild form of the disease since they have been affected by less than 10 skin tumors, which appeared after the age of 20. In addition, since our patients lack neurological dysfunction, we predicted that the disease pathology most likely resulted from coding mutations in *XPC*, *XPE* or *XPV*. Coding regions of these genes encompass 37 exons, which may be sequenced, in a single patient, via direct sequencing or NGS at similar costs. Indeed, at present primer synthesis and direct sequencing of 20 exons using internal oligonucleotides display similar costs to a single exome sequencing experiment. Furthermore, PCR amplification and Sanger sequencing of a significant number of exons may be labour-intensive.

It is important to note that, at present, downstream bioinformatic analysis of sequencing data is considered as the bottleneck of NGS techniques. Whole-genome and exome sequencing generate a considerable amount of data, which has to be quality controlled, filtered and interpreted in a biological context. This analysis implicates significant computational costs and needs a particular expertise. Although distinct bioinformatics tools have been developed in order to analyze and interpret the increasing amount of sequencing data, sensitivity and efficiency to accurately map noisy reads depends of several factors, such as the choice of single or pair-end reads, the use of long or short reads and the sequencing depth [24]. In this context, the setup of a reliable NGS platform takes a considerable period of time and may represent high costs. Additionally, it has been reported that NGS sensitivity may be low to find somatic mutations [25]. However, very recent reports indicate that this approach may display ∼90% of sensitivity to identify germline mutations [26], [27]. In the present report we have used a similar NGS approach to that described in a previous study, which led us to identify a novel gene related to pachydermoperiostosis/myelofibrosis etiology [28].

Our sequencing experiments revealed that the previously unknown *POLH* p.Y299X mutation is causative of the phenotype. Up to now, more than 50 *POLH* mutations have been associated with XPV pathogenesis [4], [10], [18], [29]–[33]. In a significant proportion of cases these mutations lead to the synthesis of mRNA molecules carrying a premature stop codon, which might be degraded by the nonsense-mediated decay (NMD) system [18]. In our case, since the premature stop codon is situated at least 50 nucleotides upstream of an exon-exon boundary, we propose that mutant mRNAs might be recognized and degraded by the NMD machinery [34]. In addition, the POLH c.897T>G mutation is predicted to generate a truncated protein of 299 residues lacking the C-terminal region of the highly conserved N-terminal domain of the protein, which comprises the enzymatic active site [31], [35], [36]. Therefore, as suggested for other nonsense biallelic mutations which truncate the N-terminal protein region, it is highly likely that the POLH- p.Y299X mutant protein lacks biological activity [31].

Taken together, our results demonstrate that whole-exome sequencing is a powerful strategy, which might be used in a near future, for XP molecular etiology determination. Furthermore, they add information on the clinical and molecular characteristics of XPV patients.
